# Integrated radiochemotherapy study of ZIF-8 coated with osteosarcoma-platelet hybrid membranes for the delivery of Dbait and Adriamycin

**DOI:** 10.3389/fbioe.2023.1147064

**Published:** 2023-02-17

**Authors:** Longhai Du, Guanghao Zhu, Yanlong Xu, Binxu Han, Yu Wang, Minhui Zhu, Yingdi Meng, Huaiwen Chen, Zuochong Yu

**Affiliations:** ^1^ Department of Orthopedics, Jinshan Hospital, Fudan University, Shanghai, China; ^2^ Department of Otolaryngology, Head and Neck Surgery, Changhai Hospital, Second Military Medical University, Shanghai, China

**Keywords:** osteosarcoma, Metal-organic framework, radiosensitizer, radiochemotherapy, hybrid membranes

## Abstract

**Introduction:** The toxic side effects of systemic high-dose chemotherapy and poor sensitivity to radiotherapy hinder the survival rate of patients with osteosarcoma (OS). Nanotechnology offers new solutions for OS treatment; however, conventional nanocarriers suffer from inadequate targeting of tumors and short *in vivo* circulation time.

**Methods:** Here, we designed a novel drug delivery system, [Dbait-ADM@ZIF-8]OPM, which uses OS-platelet hybrid membranes to encapsulate nanocarriers, to enhance the targeting and circulation time of nanocarriers, thereby enabling high enrichment of the nanocarriers in OS sites.

**Results:** In the tumor microenvironment, the pH-sensitive nanocarrier, which is the metal-organic framework ZIF-8, dissociates to release radiosensitizer Dbait and the classical chemotherapeutic agent Adriamycin for the integrated treatment of OS *via* radiotherapy and chemotherapy. Benefiting from the excellent targeting ability of the hybrid membrane and the outstanding drug loading capacity of the nanocarrier, [Dbait-ADM@ZIF-8]OPM showed potent anti-tumor effects in tumor-bearing mice with almost no significant biotoxicity.

**Conclusion:** Overall, this project is a successful exploration of the combination of radiotherapy and chemotherapy of OS treatment. Our findings solve the problems of the insensitivity of OS to radiotherapy and the toxic side effects of chemotherapy. Furthermore, this study is an expansion of the research of OS nanocarriers and provides new potential treatments for OS.

## 1 Introduction

Osteosarcoma (OS) is a common malignant tumor of mesenchymal origin in children and adolescents. Characterized by extremely high malignancy and susceptibility to recurrence and metastasis, 80%–90% of patients have systemic microfocal metastases until initial diagnosis (Chen et al., 2016). Although surgical resection combined with radiotherapy has increased the 5-year survival rate of patients with OS to 60% in the past 30 years, the lack of a major breakthrough in OS treatment has limited the survival rate of patients to under 20% once metastasis occurs (Eaton et al., 2021). Therefore, there is an urgent need to develop new treatment strategies.

Radiotherapy is a widely used treatment modality for cancer; however, OS responds poorly to radiotherapy, and conventional doses do not eradicate the tumor cells completely. Moreover, “radiation resistance” often develops over time (Schwarz et al., 2009). Ablation of OS usually requires high doses (80 Gy) of radiotherapy, with individual doses of approximately 10 Gy, which can have adverse effects. The high dose of radiation itself is a potential risk factor for OS (Pearce et al., 2012; Casali et al., 2018; Du et al., 2021; Tung et al., 2021).

Dbait is a short double-stranded DNA molecule with a free double-stranded blunt end that can effectively inhibit the repair of DNA double-strand breaks (DSBs) (Liu et al., 2017). Briefly, Dbait mimics the damaged DNA fragments in the body after radiotherapy, and recruits various DNA repair enzymes downstream, thus, preventing DSBs from being repaired in a timely and correct manner due to the lack of the required DNA repair enzymes. This improves the sensitivity of tumor tissue to radiation (Liu et al., 2017; Liu et al., 2020).

Previous studies have shown that Dbait combined with radiotherapy and chemotherapy is effective against several radiation- and chemotherapy-resistant tumors (Biau et al., 2016; Herath et al., 2016; Yao et al., 2016; Liu et al., 2017).

Adriamycin (ADM) is a first-line chemotherapeutic agent with a certain efficacy in OS treatment. Studies on current clinical outcomes have shown that lack of ADM during adjuvant chemotherapy or that reduction in the dosage of ADM during chemotherapy affects the survival rate of patients with OS (Schwartz et al., 2016). Therefore, the synergistic effect of Dbait with ADM is expected to be a new potential treatment strategy for OS.

Systemic administration of high doses of chemotherapy often leads to severe toxic side effects. Since Dbait needs to enter the cells to be effective, improving the uptake efficiency of Dbait and ADM in OS is an urgent need, and may be achieved through nanotechnology (Harrison et al., 2018). In recent years, metal-organic frameworks (MOFs) have been widely used because of their controllable size distribution, large specific surface area, adjustable backbone, and large internal space (Kreno et al., 2012). Zeolite imidazole framework-8 (ZIF-8) is a type of MOF with high chemical and thermal stability, and its large pore size can accommodate a large amount of drug. Once the drug is encapsulated by ZIF-8, its release *via* free diffusion from the small outer pores is difficult and can be achieved only through the degradation of the MOF. ZIF-8 has excellent pH sensitivity, which combined with targeting materials can be used to achieve efficient drug enrichment in OS cells (Ren et al., 2014).

The rapid development of bionanotechnology in recent years has provided a powerful tool for the development of ultra-long-cycle nanomaterials (Fang et al., 2018). Biofilms have the natural advantages of not being cleared by the immune system, non-cytotoxicity, and high bioavailability (Sun et al., 2016; Fang et al., 2018; Rao et al., 2018; Wang et al., 2018). The cell membrane markers and functions of each cell source can be preserved by hybridizing cell membranes from different cell sources. For example, Jiang et al. (2019) designed erythrocyte-cancer hybrid membranes that exhibited extended blood circulation and homotypic targeting source characteristics to specifically kill tumor cells. We selected platelet membranes (PM), which play an important role in several physiological activities such as intrinsic immune response and tumor metastasis, to hybridize OS cell membranes (OCM) so that they can highly target homologous cancer cells and form OS-Platelet hybrid membranes (OPM) (Rao et al., 2016; Sim et al., 2016; Zhu et al., 2016; Li et al., 2017). OPM both addresses the possible cytotoxicity caused by the outer hydrophobic surface of ZIF-8 and avoids phagocytic clearance by the immune system during body circulation of the drug-loaded system, thereby achieving a precise targeting effect on OS (Shearier et al., 2016).

**FIGURE 1:**
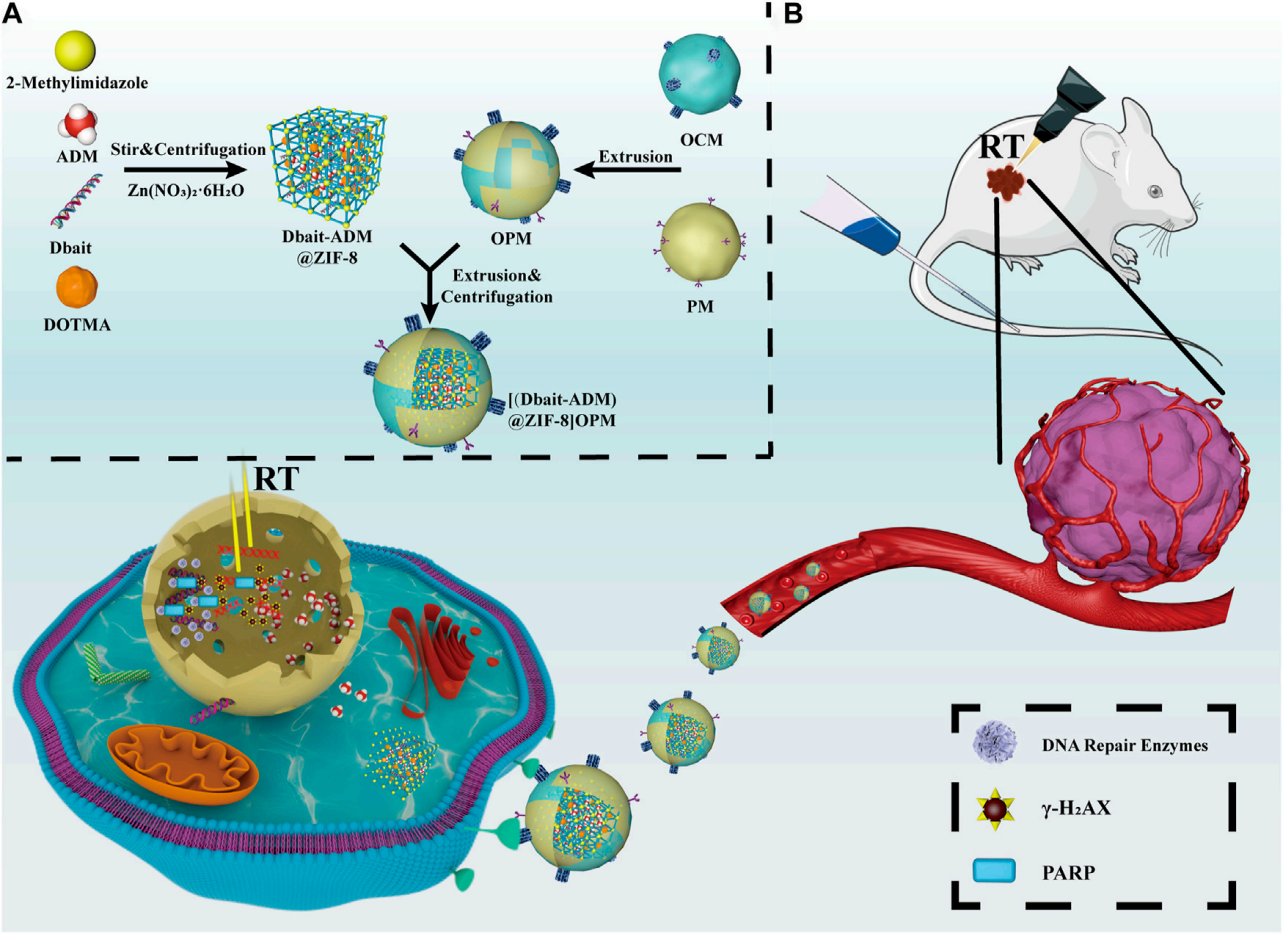
Schematic diagram of the synthesis and mechanism of action of [Dbait-ADM@ZIF-8]OPM. **(A)** Overview of the synthesis steps of [Dbait-ADM@ZIF-8]OPM. **(B)** [Dbait-ADM@ZIF-8]OPM targets SaOs-2 cells by binding to a receptor expressed on the cell membrane. Once Dbait is released into the nucleus, under RT conditions, it leads to a long-term defect in the repair of DSBs that kills OS in concert with ADM.

Therefore, based on the ability of ADM to inhibit DNA and RNA synthesis, and its possible synergy with the radiosensitizer Dbait, a novel OPM-encapsulated bionanocarrier [Dbait-ADM@ZIF-8]OPM was developed in this study to shield the clearance effect of the immune system and achieve targeting of OS cells. ZIF-8 stimulates the release of the drug for the ultimate comprehensive treatment of OS in the tumor microenvironment under low pH conditions (see Figure 1).

## 2 Materials and methods

### 2.1 Materials

2-Methylimidazole (2-MI), Zn(NO_3_)_2_·6H_2_O, and ADM were purchased from Shanghai Aladdin Bio-Chem Technology Co., Ltd. (Shanghai, China). (1R,4R,7R, 10R)-a, aʹ, aʹʹ, aʹʹʹ -Tetramethyl-1, 4, 7, 10- tetraazacyclododecane-1,4,7,10-tetraacetic acid tetrasodium salt (DOTMA) was purchased from Guidechem (Zhejiang, China). Dulbecco’s Modified Eagle Medium (DMEM), Cell Counting Kit-8 (CCK-8), GelRed, and 4′, 6-Diamidino-2-phenylin-dole (DAPI) were obtained from Jiangsu KeyGEN BioTECH Co., Ltd. (Jiangsu, China). The Annexin V-FITC/7-AAD Apoptosis Detection Kit was purchased from Sino Biological (Beijing, China). DiR, a type of near-IR fluorescent cyanine lipophilic dye, was purchased from Biotium (CA, USA). Dbait and FAM-Dbait were synthesized by Nanjing Genscript Biotechnology Co., Ltd. (Jiangsu, China), and the sequence was: 5ʹ-**GCT**GTGCCCACAACCCAGCAAACAAGCCTAGA-(H)-TCTAGG CTT​GTT​TGC​TGG​GTT​GTG​GGC​AC**AGC**-3ʹ, where (H) is a hexaethylene glycol linker and the letters that are underlined and in bold are phosphorodiamidate nucleosides.

### 2.2 Cell culture

SaOS-2 was obtained from Shanghai Fuheng Biotechnology Co., Ltd. (Shanghai, China) and was cultured in DMEM supplemented with 10% fetal bovine serum (FBS) (Gibco, United States) under humid air with 5% CO_2_ at 37°C.

### 2.3 Preparation of OPM

For OCM preparation, 1 × 10^8^ SaOS-2 cells were digested down and the liquid was aspirated after resuspension once in phosphate-buffered saline (PBS); followed by the addition of an isolation buffer containing 225 mM mannitol, 75 mM sucrose, 0.5 mM EDTA, and 30 mM Tris-HCl and 1% (v/v) protease inhibitor mixture. The suspension was sonicated in an ice bath with a probe sonicator (30 W, amplitude 3 min, on 2 s, off 3 s). The suspension was centrifuged at 800 ×g for 10 min at 4°C to remove cell debris. The supernatant was collected and centrifuged at 10,000 ×g (4°C, 30 min). Subsequently, the mitochondria were discarded, and the membrane was stored in the precipitate at −80°C as a backup.

Platelet hybrid membranes (PM) were obtained from a pure platelet suspension (Jinshan Hospital Blood Bank, Shanghai, China). After centrifugation at 800 ×g for 20 min, the supernatant was discarded, and the platelets were suspended in a PBS buffer containing 1 mmol/L EDTA and protease inhibitor to prepare a platelet suspension. The above suspension was frozen at −80°C and thawed at 25°C. This was repeated thrice, followed by centrifugation at 4000 ×g for 3 min at room temperature to obtain pelleted platelet membranes, which were washed thrice with PBS containing protease inhibitor and dispersed in sterile water. Finally, the suspension was sonicated at 42 kHz and 100 W for 5 min to obtain platelet membranes, which were stored at −80°C.

The protein concentrations of the PM and OCM suspensions were measured separately using the Beyotime BCA Protein Assay Kit (Beyotime, China). The extracted PM and OCM were mixed at a total protein ratio of 1:1 (whose protein ratio represents the membrane ratio). The mixture was placed in an Avanti Mini-Extruder (Avanti Polar Lipids, United States) and extruded back and forth 20 times using 400 nm and 200 nm polycarbonate porous membranes. The OPM suspension was then formed *via* centrifugation at 15,000 rpm for 15 min at 4°C and the precipitate was stored at −80°C.

### 2.3 Preparation of Dbait-ADM@ZIF-8

The ADM and Dbait drug stock solution was prepared using 10 mg/mL ADM and 5 nmol/100 μl Dbait.

Nanocarriers with different contents of Dbait were prepared according to N/P ratios of 4:1, 8:1, 12:1, 16:1, and 20:1. The corresponding nanocarriers were prepared using the one-pot method. 2-Methylimidazole (1.1 g in 10 mL DEPC water), 2.20 mL of ADM, 200 μl (100 mg/mL) DOTMA, and Dbait were mixed. Subsequently, 10 mL of Zn(NO_3_)_2_·6H_2_O (24 mM) was injected rapidly into the mixture under continuous stirring for another 5 min. Finally, the product was collected *via* centrifugation at 13,000 rpm for 10 min. The unreacted reactants and the drug adsorbed on the surface of ZIF-8 with a weak force were removed by washing thrice with water. The prepared Dbait-ADM@ZIF-8 was dried and stored at 4°C for further use. All other types of nanocarriers were prepared following the above steps.

### 2.4 Agarose gel electrophoresis

The ability of nanocarriers to encapsulate Dbait was determined using agarose gel electrophoresis. A 3.0% agarose gel was prepared and cooled to 40°C, and GelRed was added. Subsequently, the sample was loaded. Electrophoresis at 100 V was performed for 30 min and the gel was observed using a UV projector and photographed.

The stability of Dbait-ADM@ZIF-8 was assessed after N/P refinement (8:1, 10:1, 12:1) using the DNase I digestion and anion replacement methods. DNase I digestion was performed by adding a DNase I solution (Beyotime Biotechnology, Shanghai, China) to freshly configured nanoparticles and incubating them at 37°C for 2 h. The enzyme reaction was terminated using EDTA and 3% agarose gel electrophoresis was performed under the same conditions. The anion replacement method was performed by adding anionic heparin solution to freshly prepared nanoparticles and incubating for 20 min. Agarose gel electrophoresis was performed again under the same conditions.

### 2.5 Synthesis of [Dbait-ADM@ZIF-8]OPM

The OPM suspension was added to Dbait-ADM@ZIF-8 at a ratio of 8:1. Subsequently, the mixture was extruded on the 200 nm polycarbonate porous membranes eleven times using the Avanti Mini-Extruder, and then centrifuged to remove the empty hybrid membrane vesicles.

### 2.6 Characterization of [Dbait-ADM@ZIF-8]OPM

Western blot (WB) analysis of OCM, PM, OPM, and [Dbait-ADM@ZIF-8]OPM was performed for the characterization of the OCM marker EGFR (Abcam, United Kingdom) and PM marker CD47 (Abcam, United Kingdom). Transmission electron microscopy (TEM) images of [Dbait-ADM@ZIF-8]OPM were obtained using a Talos L120C (Thermo Fisher Scientific Inc., MA, United States). The hydrodynamic diameters and zeta potential were measured using a Zetasizer Nano Series system (Nano ZS90, Malvern Panalytical Ltd., United Kingdom) at 25°C. The powder XRD pattern was captured using a PANalytical X'Pert Pro MPD (Malvern Panalytical Ltd., UK) with a Cu Kα radiation source (2θ, 5°–60°).

### 2.7 Drug loading and release analysis

To calculate the contents of Dbait and ADM in [Dbait-ADM@ZIF-8]OPM, the average absorbance of different concentrations of Dbait and ADM at 260 nm and 488 nm was determined using Varioskan Flash (Thermo Fisher Scientific Inc., MA, United States). The standard curves of Dbait and ADM were established.

[Dbait-ADM@ZIF-8]OPM (15 mg) was completely dissolved in 1 mL of PBS at pH 1. Subsequently, the absorbance was determined using Varioskan Flash, and the concentrations of Dbait and ADM in the sample solution were deduced from the standard curve. The drug loading was calculated. Loading capacity (%) = (A−A_1_)/A_2_ × 100, where A represents the initial amount of Dbait and ADM added, A_1_ represents the content of Dbait and ADM in the supernatant, and A_2_ represents the content of [Dbait-ADM@ZIF-8]OPM.

[Dbait-ADM@ZIF-8]OPM (150 mg) was dissolved in 10 mL of PBS at pH 5 and 7. The samples were then placed on a 37°C shaker and the concentrations of Dbait and ADM were determined using Varioskan Flash at different time points.

### 2.8 Cellular uptake and localization of [Dbait-ADM@ZIF-8]OPM

Cellular uptake of [Dbait-ADM@ZIF-8]OPM was tested using a BD FACSCalibur flow cytometer. Free FAM-Dbait, (FAM-Dbait)-ADM@ZIF-8, and [(FAM-Dbait)-ADM@ZIF-8]OPM were incubated with SaOS-2 cells for 5 h. The cells were washed twice with PBS, digested with trypsin solution, and analyzed using a BD Calibur.

The targeting ability of [Dbait-ADM@ZIF-8]OPM was detected using a fluorescence microscope (Olympus IX73, Japan). Briefly, the above materials were incubated with SaOS-2 cells for 5 h. The cells were fixed using 4% paraformaldehyde at room temperature for 15 min and the nuclei were stained with DAPI at 25°C for 10 min in the dark.

To determine the localization of [Dbait-ADM@ZIF-8]OPM, SaOS-2 cells (5 × 10^4^ cells/well) were inoculated in 35-mm confocal culture dishes and incubated for 12 h. Freshly prepared [(FAM-Dbait)-ADM@ZIF-8]OPM was transfected and incubated for another 1, 3, and 5 h, respectively. The medium was removed, and the cells were co-incubated with configured Lyso-Tracker Red (Beyotime, China) for 40 min at 37°C. The cells were fixed using 4% paraformaldehyde for 15 min at room temperature and their nuclei were stained with DAPI at 25°C for 10 min in the dark. The cells were photographed using a confocal laser scanning microscope (CLSM, Olympus FV3000, Japan).

### 2.10 Radiosensitization effects of [Dbait-ADM@ZIF-8]OPM

#### 2.10.1 CCK-8 assay

The CCK-8 method was used to compare the effects of different treatments on cell survival. Briefly, SaOS-2 cells (1 × 10^4^ cells/well) were inoculated on 96-well plates with five replicate wells per group. The cells were treated with PBS, Dbait@ZIF-8, ADM@ZIF-8, Dbait-ADM@ZIF-8, and [Dbait-ADM@ZIF-8]OPM (containing 0.258 μg ADM, 0.02 μg Dbait) 12 h later. Subsequently, 200 μl of new culture medium was added to each well. The cells were incubated for another 24 h. Thereafter, 20 μl CCK-8 reagent was added to each well and the cells were incubated for 1 h. The absorbance of each well was measured using Varioskan Flash at 450 nm. Cell viability was calculated as OD_450test_/OD_450control_.

#### 2.10.2 Colony formation assay

A colony formation assay was used to compare the effect of different treatments on cell proliferation. Briefly, cells were seeded in 6-well plates at the appropriate cell density and cultured routinely. The cells were treated with PBS, Dbait@ZIF-8, ADM@ZIF-8, Dbait-ADM@ZIF-8, and [Dbait-ADM@ZIF-8]OPM (containing 2.58 μg ADM, 0.2 μg Dbait) 12 h later. After 5 h, the cells were treated with 0 Gy, 2 Gy, 4 Gy, 6 Gy, and 8 Gy (0.3 Gy/min) doses of irradiation (IR). After approximately 14 days, the cells were fixed with 4% paraformaldehyde, followed by staining with crystal violet (Beyotime, China). All colonies of more than 50 cells were counted and cell survival curves were obtained using GraphPad Prism software 8.0.

#### 2.10.3 Cell apoptosis assay

The apoptosis rate was measured using Annexin V-FITC/7-AAD Apoptosis Detection Kit. Briefly, SaOS-2 cells at a density of 3 × 10^5^ were inoculated in 6-well plates. The cells were treated as described above in the WB 24 h later. After 5 h, the cells were irradiated with 2 Gy (0.3 Gy/min). The cells were collected 48 h later, washed twice with pre-cooled PBS at 4°C, centrifuged to remove the supernatant, and then resuspended in 1 × Binding Buffer. The cell suspension was transferred to a flow tube, Annexin V-FITC and 7-AAD were added, and the mixture was gently mixed and incubated at room temperature (25°C) for 15 min in the dark. The suspension was tested using a BD FACSCalibur flow cytometer within 1 h after termination of the reaction.

#### 2.10.4 Western blot

Cells that grew logarithmically were treated with PBS, radiotherapy (RT), RT + Dbait@ZIF-8, RT + ADM@ZIF-8, RT + Dbait-ADM@ZIF-8, and RT+[Dbait-ADM@ZIF-8]OPM (2 Gy, 0.3 Gy/min). The cells were harvested 48 h later and incubated in RIPA Lysis Buffer (Epizyme, China) containing protease and phosphatase inhibitors (Beyotime, China) for 30 min on ice. The cells were centrifuged at 12,000 rpm for 10 min at 4°C. The supernatant was collected, and the protein concentration was detected using the BCA Protein Assay Kit. After denaturation, the proteins were separated *via* 4%–20% SurePAGE gel (GenScript, China) and electro-transferred to polyvinylidene fluoride membranes (Millipore, United States). Subsequently, the membranes were blocked with 5% BSA and incubated overnight at 4°C with the corresponding primary antibodies: γ-H2AX (Cell Signalling Technology, United States), PARP (Cell Signalling Technology, United States), and GAPDH (Beyotime, China) (dilution ratio = 1:1000). The membranes were then incubated with horseradish peroxidase (HRP)-conjugated secondary antibody (Beyotime, China) for 1 h at room temperature. Finally, the immunoreactive bands were developed with a chemiluminescent HRP substrate (Beyotime, China) (dilution ratio = 1:1000) and photographed using a chemiluminescent image system (GE ImageQuantLAS4000, United States). This was repeated thrice, and the intensity of the bands was analyzed using ImageJ software.

### 2.11 Animal model

Five-week-old female BALB/c nude mice were purchased from Vital River Laboratory Animal Technology Co., Ltd. (China). All the following operations were performed according to the guidelines of the Animal Specialty Committee of the Second Military Medical University (Shanghai, China). All the mice were raised under standard conditions and were free of pathogens. In short, 5 × 10^−6^ logarithmic SaOS-2 cells were mixed with Matrigel (CORNING, United States) at 1:1 and subcutaneously injected into BALB/c nude mice. The above operations were performed on ice. In addition, if the weight loss exceeded 10% or the tumor size exceeded 2 cm^3^ after the injection of the appropriate medication, the mice were executed.

### 2.12 *In Vivo* tissue distribution of [Dbait-ADM@ZIF-8]OPM

Balb/c nude mice with tumor volumes up to 500 mm^3^ were randomly divided into three groups with four mice in each group and intravenously injected with PBS, Dir-(Dbait-ADM)@ZIF-8, and [Dir- (Dbait-ADM)@ZIF-8]OPM (10 μg dir/rat). Once the mice were anesthetized by intraperitoneal injection of 1% pentobarbital sodium solution at a dose of 0.1 mL per 10 g, the *in vivo* images were observed using an *in vivo* imaging system (excitation/emission wavelength: 780/810 nm, direct fluorescence), and the images were recorded at different time points with a built-in charge-coupled device camera. After 24 h, the mice were killed using carbon dioxide, and the excised organs and tumors were imaged and quantitatively analyzed. For the *in vivo* uptake studies, tumors were collected from sacrificed mice, immediately placed in optimum cutting temperature compound medium, and quickly frozen in liquid nitrogen. Frozen sections were cut into 10-μm sections and fixed with acetone at −20°C. After cleaning with PBS, the sections were subjected to DAPI re-staining and were observed under a fluorescence microscope (excitation/emission wavelength of ADM: 480/593 nm).

### 2.13 *In Vivo* antitumor activity

Thirty-six Balb/c nude mice with a tumor volume of approximately 100 mm^3^ were randomly divided into six groups: 1) PBS; 2) RT; (3) RT + Dbait@ZIF-8; 4) RT + ADM@ZIF-8; 5) RT + Dbait-ADM@ZIF-8; 6) RT+[Dbait-ADM@ZIF-8]OPM. They were injected with drugs (3 mg ADM/kg, 0.233 mg Dbait/kg) through the tail vein. After 5 h, only the tumor was irradiated with 2 Gy (0.3 Gy/min), and the rest of the mice were shielded using lead. The treatment was performed every 3 days. At the same time, the two vertical diameters of the tumor were measured using calipers and the mouse was weighed. The tumor volume was calculated using formula V = 0.5 × a × b^2^, where a and b represent the larger and smaller diameters, respectively. When the tumor volume reached 2000 mm^3^ or at 30 days after administration, the treatment was terminated, and histological samples were collected for follow-up analysis.

### 2.14 Toxicity evaluation

Specimens collected for treatment endpoints, i.e., heart, liver, spleen, lung, and kidney samples, were fixed with 4% paraformaldehyde and subjected to hematoxylin-eosin (H&E) staining.

### 2.15 Western blot

After 48 h of the above treatment, four rats in each group were randomly executed and the excised tumors were subjected to WB assay. Briefly, tumor tissues were ground in a mortar with liquid nitrogen, protein extraction solution was added, and the mixture ice-bathed for 20 min. The mixed liquid was centrifuged at 14,000 rpm for 20 min at 4°C, and the supernatant was aspirated and stored at −80°C. The aspirated supernatant was whole cell protein. The subsequent steps were the same as those in the previous WB experiments.

### 2.16 Statistical analysis

All experiments were performed at least thrice. Quantitative data were expressed as the mean and standard deviation (SD), and statistical analysis was performed using GraphPad Prism 8.0. For comparisons between two groups, the Student’s t-test or Chi-square test was used. *p < 0.05; **p < 0.01; ***p < 0.001; ****p < 0.0001.

## 3 Results

### 3.1 Characterization of [Dbait-ADM@ZIF-8]OPM

**FIGURE 2:**
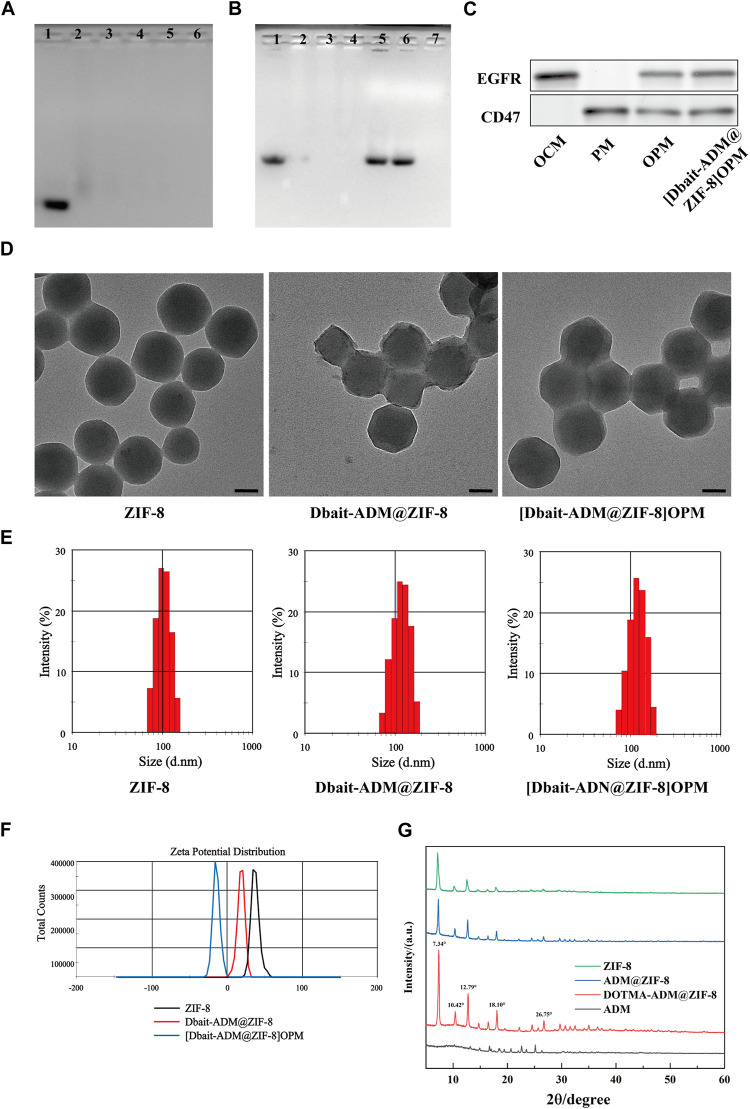
Characterization of [Dbait-ADM@ZIF-8]OPM. **(A)** Agarose gel electrophoresis was used to determine the stability of the nanocarriers at different N/P ratios (1–6: Dbait = 1:4, 1:8, 1:12, 1:16, 1:20) and **(B)** DNase I digestion and anion replacement (1: Dbait; 2–4: Dnase I treated = 1:8, 1:10, 1:12; 5–7: Heparin treated = 1:8, 1:10, 1:12) nanocarrier stability. **(C)** Western blot analysis of EGFR and CD47 markers originating from OCM, PM, and OPM, [Dbait-ADM@ZIF-8]OPM. **(D)** TEM images of ZIF-8, Dbait-ADM@ZIF-8, and [Dbait-ADM@ZIF-8]OPM. Scale bar, 50 nm. **(E)** Particle size of ZIF-8, Dbait-ADM@ZIF-8, [Dbait-ADM@ZIF-8]OPM. **(F)** Zeta potential of ZIF-8, Dbait-ADM@ZIF-8, [Dbait-ADM@ZIF-8]OPM. **(G)** XRD of ZIF-8, ADM@ZIF-8, DOTMA-ADM@ZIF-8 and ADM.

The results of the agarose gel electrophoresis of the DNA binding affinity of Dbait-ADM@ZIF-8 (Figure 2A) showed that Dbait was retained in the nanocarrier after N/P refinement at 8:1. Stability evaluations using the DNase I digestion and anion replacement methods showed that neither precipitated at an N/P of 12:1 (Figure 2B); therefore, this ratio was chosen as a parameter for the synthesized material. Next, examination of the tumor cell membranes, using WB analysis after nanoparticle encapsulation, detected the proteins EGFR and CD47, specific for OCM and PLM, on the hybrid membrane and [Dbait-ADM@ZIF-8]OPM (Figure 2C). This indicates that the hybrid membrane had attached to the nanocarrier surface. The TEM images (Figure 2D) showed that the average size of the prepared [Dbait-ADM@ZIF-8]OPM was 128 ± 4 nm and was characterized by dynamic light scattering. The encapsulation of the cell membrane on the nanocarrier surface was determined by comparing the hydration dynamics diameter and zeta potential of the nanoparticles before and after encapsulation. We found that the diameter of Dbait-ADM@ZIF-8 increased from 115.32 ± 6.42 nm to 125.57 ± 4.68 nm after encapsulation of the hybridized membrane (Figure 2E), which was consistent with the TEM images, and its zeta potential changed from 16.6 ± 5.39 mV to −12.3 ± 5.81 mV (Figure 2F). These changes may be explained by the fact that the cell membrane has a certain thickness and carries a less negative surface charge, further indicating that the tumor membrane has been encapsulated on the surface of the nanocarrier. The XRD results showed that the crystal structure of ZIF-8 obtained from the preparation in this study was the same as reported (Figure 2G), and the loading of the drug did not notably disrupt its structural integrity. Therefore, it was feasible for us to synthesize these nanocarriers using a simple and convenient “one-pot” method (Feng et al., 2020).

### 3.2 Drug loading and release analysis

**FIGURE 3:**
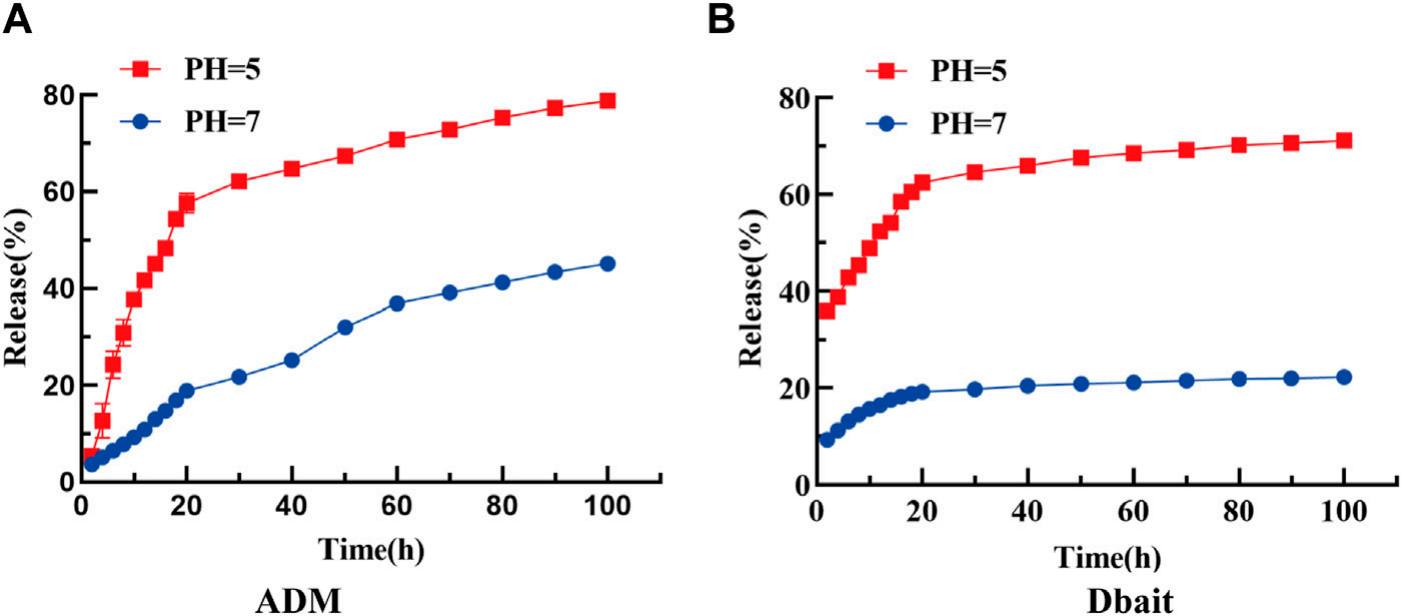
Cumulative release rates of **(A)** ADM and **(B)** Dbait in PBS at pH = 5, 7.

The loading efficiency of Dbait and ADM in [Dbait-ADM@ZIF-8]OPM was 0.73% and 9.42%, respectively, and the efficient encapsulation rate of Dbait was attributed to the positive charge provided by DOTMA (Supplementary Figures S1A,B show the standard curves of ADM and Dbait, respectively). The abundant loading capacity facilitated exploring their efficacy in the tumor microenvironment. Furthermore, the release rates of Dbait and ADM were 45.08% and 22.25% in PBS at pH 7.0, whereas, their release rates were 78.77% and 71.13% in PBS at pH 5.0, respectively. These results suggest that ZIF-8 remains stable under neutral conditions and is readily degraded under acidic stimuli, thus releasing the encapsulated payload specifically in the acidic tumor microenvironment (Figures 3A,B). Furthermore, ADM and Dbait do not interact during the slow release process in the [Dbait-ADM@ZIF-8]OPM system (Supplementary Figure. S1C).

### 3.3 *In Vitro* targeting capability of [Dbait-ADM@ZIF-8]OPM

**FIGURE 4:**
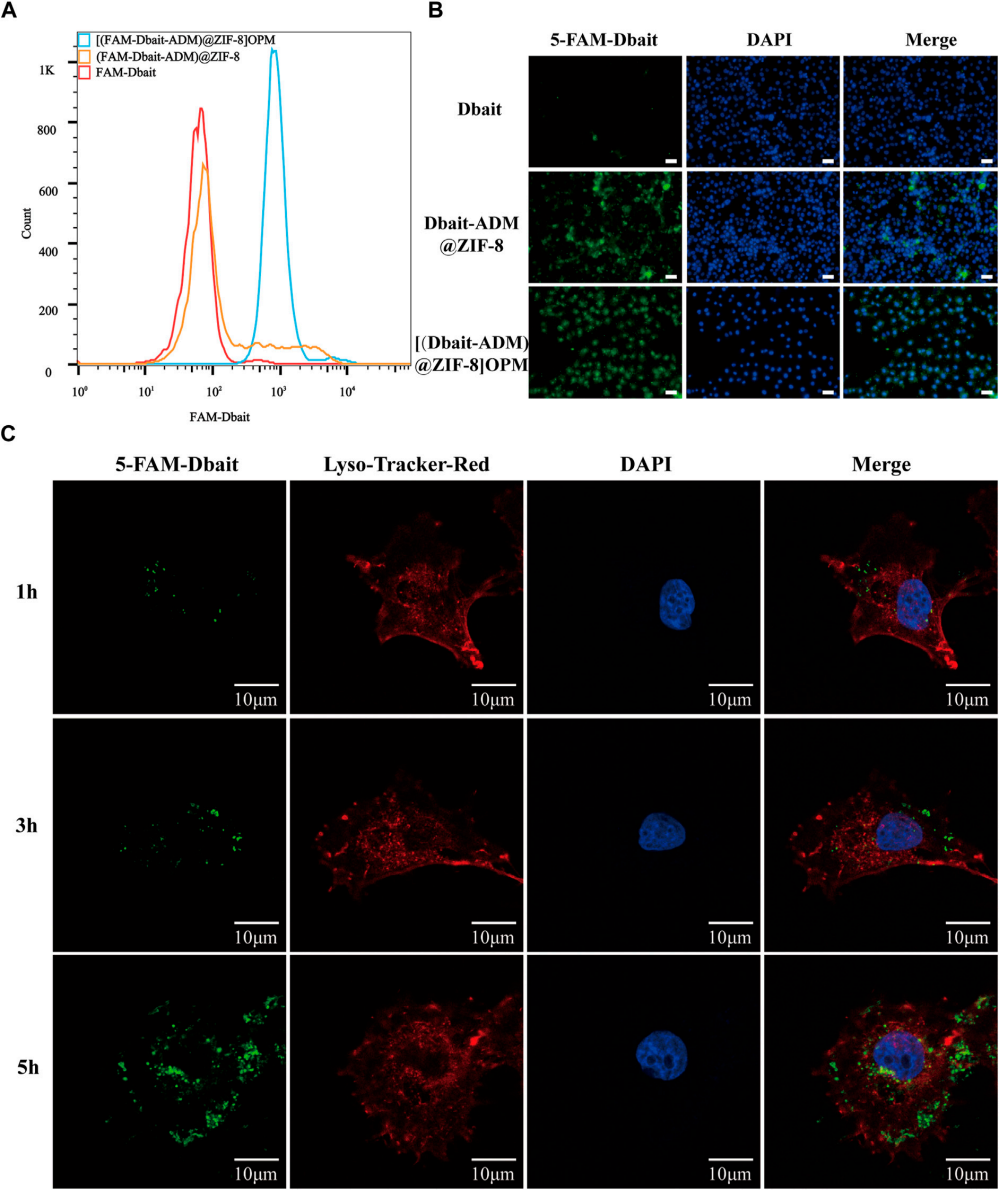
*In vitro* targeting capability of [Dbait-ADM@ZIF-8]OPM. **(A)** Cellular uptake of free Dbait, Dbait-ADM@ZIF-8, [Dbait-ADM@ZIF-8]OPM was analyzed by flow cytometry. **(B)** Entry of free Dbait, Dbait-ADM@ZIF-8, [Dbait-ADM@ZIF-8]OPM into the cells was observed by fluorescence microscopy. Scale bar, 50 nm. **(C)** CLSM images of SaOS-2 cells cultured with [Dbait-ADM@ZIF-8]OPM for 1, 3, and 5 h. Dbait was labeled with 5-FAM (green), late endosomes and lysosomes were stained with Lyso-Tracker Red (red) and nuclei were stained with DAPI (blue). Scale bar, 10 μm.

The uptake of bare Dbait, Dbait-ADM@ZIF-8, and [Dbait-ADM@ZIF-8]OPM by SaOS-2 cells were analyzed using flow cytometry. The fluorescence intensity of the [Dbait-ADM@ZIF-8]OPM-treated group was much higher than that of the free Dbait-treated group compared with the bare Dbait, the no-membrane-coated group, indicating that the hybrid membrane provided very good targeting ability (Figure 4A). Using fluorescence microscopy to observe the above three treatment groups at the microscopic level, we observed that the [Dbait-ADM@ZIF-8]OPM-treated group showed the same results as the flow pattern (Figure 4B). We observed the localization of [Dbait-ADM@ZIF-8]OPM in the cells *via in vitro* staining of endosomes and lysosomes with Lyso-TrackerRed (Figure 4C). At 3 h, a strong green and red fluorescence were observed to coexist. After 5 h of incubation, the green fluorescence overlapped with the blue fluorescence, indicating that Dbait had been stably delivered into the nucleus. This confirmed that Dbait exerts radiosensitizing effects.

### 3.4 *In Vitro* antitumor efficacy of [Dbait-ADM@ZIF-8]OPM

#### 3.4.1 CCK-8 assay

**FIGURE 5:**
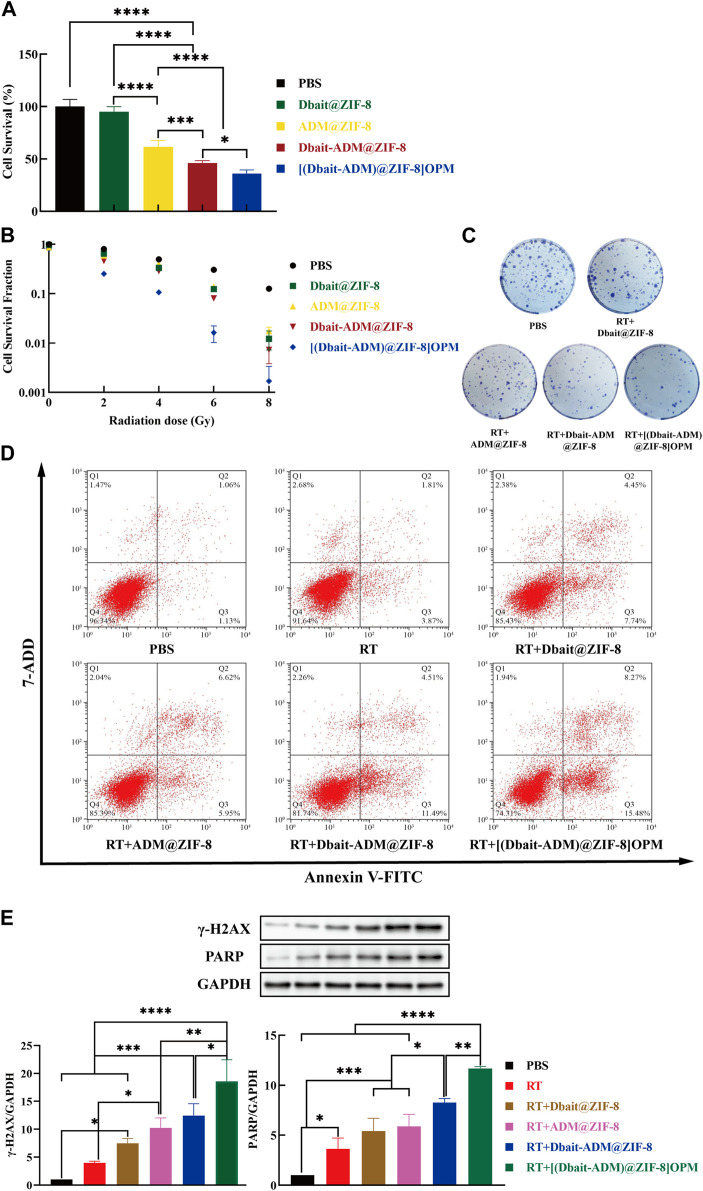
*In vitro* antitumor efficacy of [Dbait-ADM@ZIF-8]OPM. **(A)** The effect of PBS, Dbait@ZIF-8, ADM@ZIF-8, Dbait-ADM@ZIF-8, and [Dbait-ADM@ZIF-8] OPM on the survival of SaOS-2 cells was detected by CCK-8 assay. **(B)** Under the condition of different doses of RT, the effect of each group on the proliferation of SaOS-2 cells was detected by colony formation assay. **(C)** The colony formation of SaOS-2 cells in different treatment groups under 2 Gy conditions. **(D)** PBS, Dbait@ZIF-8, ADM@ZIF-8, Dbait-ADM@ZIF-8, and [Dbait-ADM@ZIF-8] OPM induced apoptosis in SaOS-2 cells induced by 2 Gy RT. **(E)**Western blot analysis of γ-H2AX and PARP protein levels in SaOS-2 cells.

The cytotoxicity of [Dbait-ADM@ZIF-8]OPM was investigated in SaOS-2 cells. There was no significant difference between the control group and the Dbait@ZIF-8 group, indicating that the nanocarriers ZIF-8 and Dbait were essentially non-cytotoxic in the absence of RT (Figure 5A). The [Dbait-ADM@ZIF-8] OPM group had the lowest cell survival rate, which was significantly different from all other treatment groups. Compared with the Dbait-ADM@ZIF-8 group, the difference in survival should stem from the targeting capability provided by the hybrid membrane, allowing more drug enrichment within the cells to achieve enhanced efficacy.

#### 3.4.2 Colony formation assay

The colony formation assay is an *in vitro* cell survival assay that detects the sensitivity of cells to killing factors based on the ability of single cells to form colonies (Jiao et al., 2019). Therefore, the colony formation assay was performed to determine the combined efficacy of [Dbait-ADM@ZIF-8]OPM by inhibiting cell growth. In the absence of RT, the results were similar to those of the CCK-8 assay (Figure 5B), where ZIF-8 and Dbait were essentially noncytotoxic, and the hybrid membrane increased the intracellular drug concentration of ADM. With the increase of irradiation dose, the cell proliferation efficiency of [Dbait-ADM@ZIF-8]OPM was significantly lower than that of other dose groups (*p* < 0.05). The cell proliferation efficiency at 2 and 4 Gy was only 25.17% and 10.65%, respectively, suggesting that [Dbait-ADM@ZIF-8]OPM could effectively enhance the radiosensitization of cells at low doses of RT. Since high-dose irradiation can damage both normal brain function and cognitive function, we chose 2 Gy for follow-up experiments (Figure 5C) (McDuff et al., 2013).

#### 3.4.3 Cell apoptosis assay

Apoptosis is an important indicator of the DNA damage caused by ionizing radiation. To avoid the effect of ADM autofluorescence, the AnnexinV-FITC/7-AAD staining assay was performed using flow cytometry. In the control group, SaOS-2 cells showed early and late apoptosis levels of 1.06% and 1.13%, respectively (Figure 5D). This type of apoptosis is normal for untreated cells as they are likely to have undergone apoptosis under these growth conditions. Cells treated with [Dbait-ADM@ZIF-8]OPM showed the maximum amount of apoptosis, i.e., the sum of early and late apoptosis rates was 23.75%. This confirms that [Dbait-ADM@ZIF-8]OPM has a superior ability to induce apoptosis.

#### 3.4.4 Western blot analysis

In higher eukaryotic cells, the presence of γ-H2AX in the nucleus is commonly used as an indicator of the presence of DSBs (Luczak and Zhitkovich, 2018). Therefore, we also investigated other DNA damage-related proteins, such as PARP (Figure 5E). The levels of γ-H2AX and PARP were significantly increased in all groups compared to those in the control and RT groups. This indicated that the production of DSBs in SaOS-2 cells was significant after treatment. The synergistic effect played by the integrated radiotherapy treatment of Dbait and ADM (*p* < 0.05) was verified at the molecular level. Furthermore, [Dbait-ADM@ZIF-8]OPM allowed an increased concentration of the drug in the cells compared with that in the cell-free membrane treatment group, which also demonstrated that the hybrid membrane enhances the targeting ability of the nanocarriers.

### 3.5 *In vivo* tissue distribution of [Dbait-ADM@ZIF-8]OPM

**FIGURE 6:**
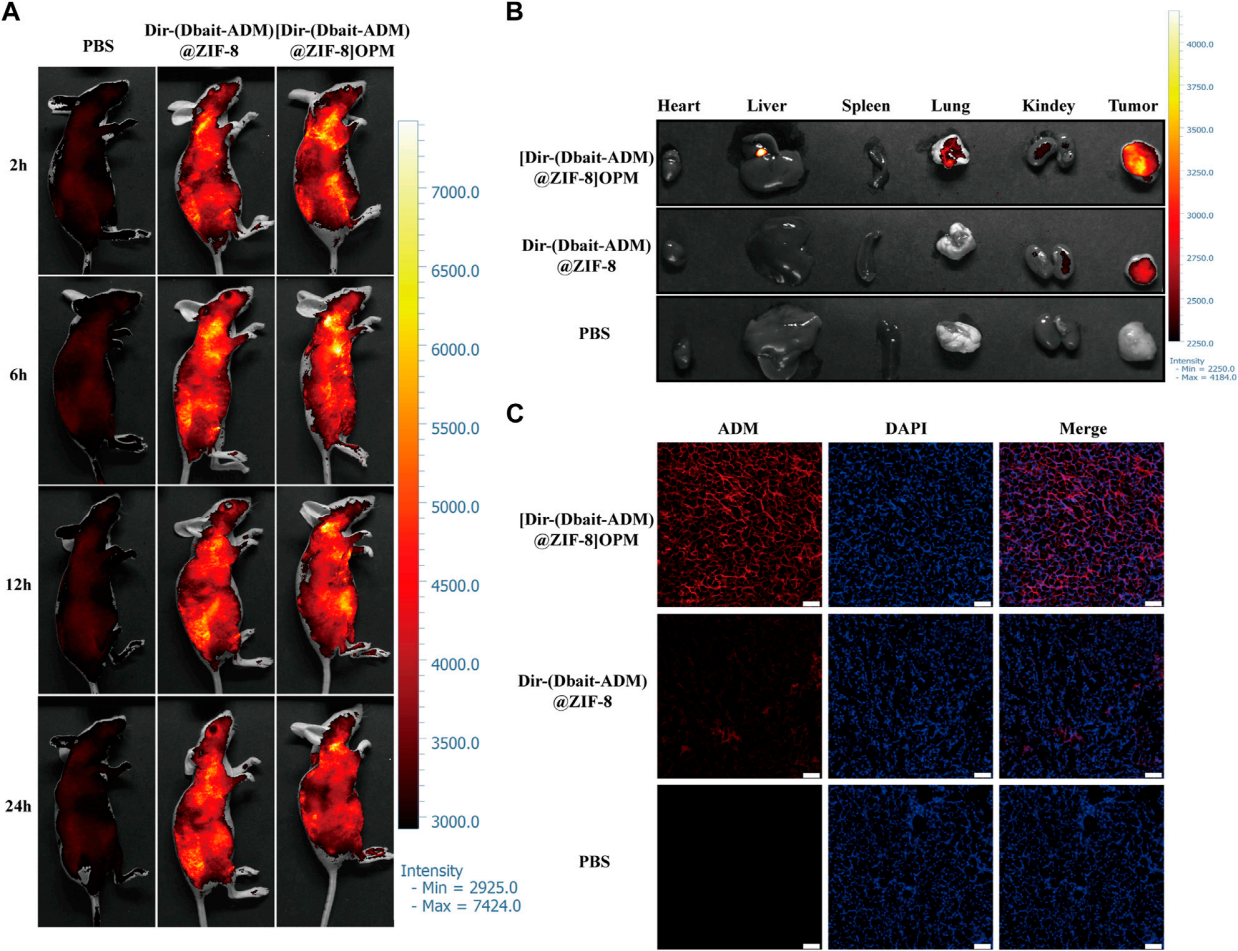
*In vivo* tissue distribution of [Dbait-ADM@ZIF-8]OPM. SaOS-2 tumor-bearing Balb/c nude mice were injected with PBS, Dir-(Dbait-ADM)@ZIF-8, and [Dir-(Dbait-ADM)@ZIF-8]OPM, respectively, in the tail vein. **(A)** *In vivo* images of mice at 2, 6, 12, and 24 h after treatment in each of the above groups. **(B)** *In vitro* images of tumors and other organs at 24 h after treatment. **(C)** *In vivo* uptake studies. Frozen sections of excised tumors were stained with DAPI and observed *via* fluorescence microscopy. Scale bar, 50 μm.

The tissue distribution of [Dbait-ADM@ZIF-8]OPM loaded with Dir was assessed subcutaneously in mice suffering from OS. Figure 6A shows the real-time distribution and tumor accumulation of PBS, Dir-(Dbait-ADM)@ZIF-8, and [Dir-(Dbait-ADM)@ZIF-8]OPM after tail vein injection at 2, 6, 12, and 24 h. Dir-(Dbait-ADM)@ZIF-8 and [Dir-(Dbait-ADM)@ZIF- 8]OPM accumulated in the tumor within 2 h, but the tumor site accumulation of [Dir-(Dbait-ADM)@ZIF-8]OPM was significantly higher than that of Dir-(Dbait-ADM)@ZIF-8. The tumor fluorescence in [Dir-(Dbait-ADM)@ZIF-8]OPM remained very pronounced over time, even at 24 h, whereas the tumor fluorescence in Dir-(Dbait-ADM)@ZIF-8 was very faint and had largely disappeared at 24 h.

To observe the fluorescence signal, the tumor and major organs were collected *via* excision at 24 h after injection. The *ex vivo* fluorescence images of the excised tumor further showed that [Dir-(Dbait-ADM)@ZIF-8]OPM accumulation was significantly higher in the tumor than that in the no-hybrid membrane treated group (Figure 6B). There was a significant accumulation of nanoparticles in the liver, whereas no significant fluorescence uptake was observed in the heart and spleen, which showed only moderate background signals.

To study the binding and internalization of nanoparticles *in vivo*, fluorescence microscopy observations were performed of the uptake of nanomaterials in OS tumor sections. Figure 6C shows a significant accumulation of [Dir-(Dbait-ADM)@ZIF-8]OPM in the tumor tissue, which is consistent with the *in vivo* imaging data. All of the above results demonstrate that the hybridized membranes enable the nanocarriers to obtain excellent targeting properties.

### 3.6 *In Vivo* inhibition of subcutaneous tumor growth by [Dbait-ADM@ZIF-8]OPM

**FIGURE 7:**
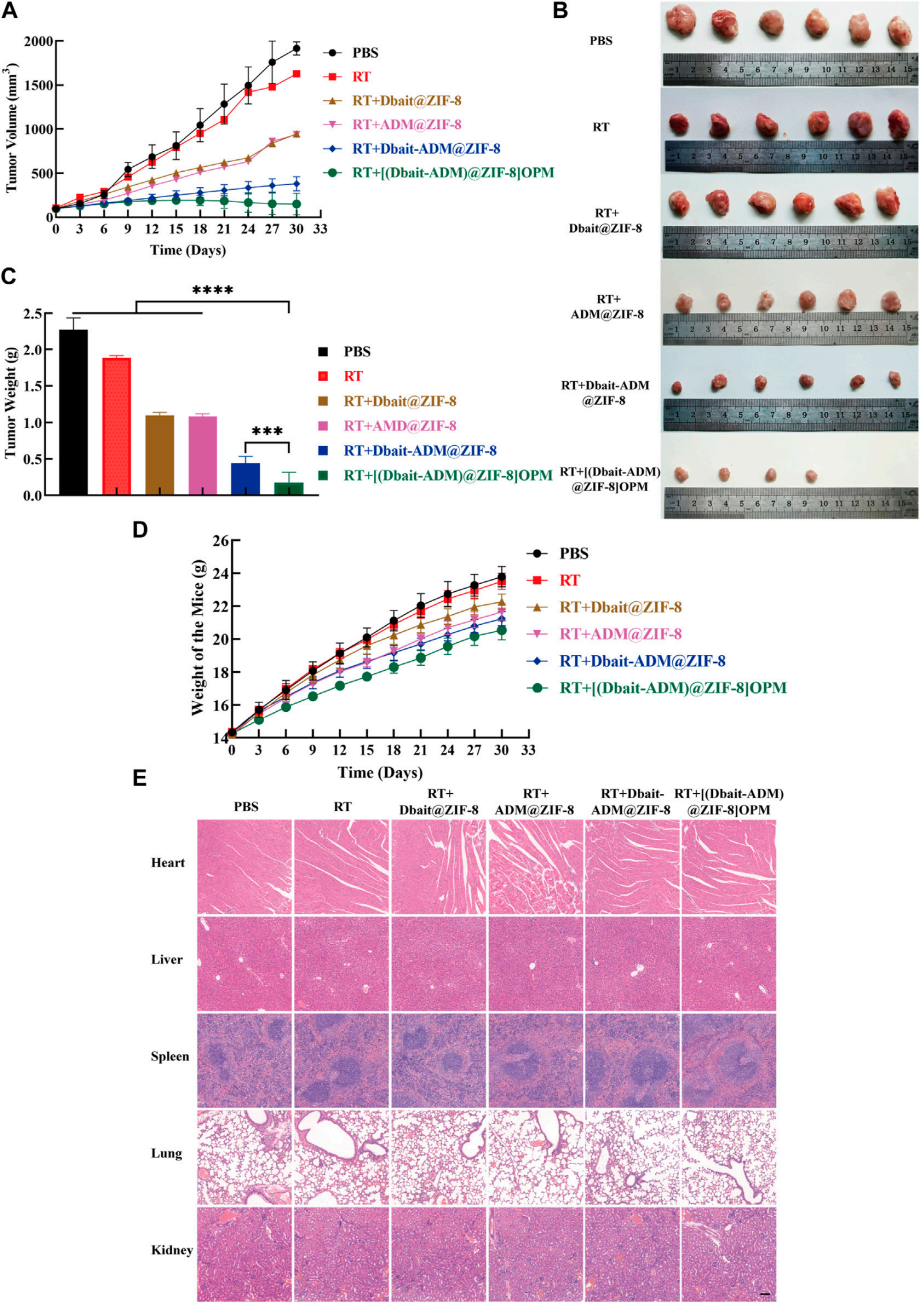
Antitumor activity *in vivo*. **(A)**The tumor growth curve. **(B)** Images of excised tumors of each group at the endpoint. **(C)** Weight of the excised tumors at the endpoint. **(D)** The weight change of the mice during the treatment. The body weight of the mice was monitored (n = 6). **(E)** H&E histological staining of nude mice organs after the above treatment. Scale bar, 50 μm.

The above-mentioned products were administered to nude mice carrying OS tumors. Measuring the changes in tumor volume (Figure 7A) showed that PBS did not show any anti-tumor effect. On day 27, a nude mouse was killed because the tumor volume exceeded 2000 mm^3^. The curative effect of the simple RT-treated group was very limited, and the tumor progressed rapidly. By the end of the experiment, Dbait@ZIF-8 and ADM@ZIF-8 reduced the tumor volume by only 50.62% and 51.07%, respectively. Compared with the initial tumor volume (100 mm^3^), the tumor volume of mice treated with Dbait-ADM@ZIF-8 increased by 2.8 times. Notably, the tumor volume of mice treated with [Dbait-ADM@ZIF-8]OPM increased by only 0.5 times and, in two mice, no tumor could be measured or observed anatomically.

The weight of the resected tumors was measured (Figures 7B,C). The mean tumor weight of the [Dbait-ADM@ZIF-8]OPM treatment group was significantly lower than that of the other groups ([Dbait-ADM@ZIF-8]OPM = 0.17 g, Dbait-ADM@ZIF-8 = 0.44 g, ADM@ZIF-8 = 1.09 g, Dbait@ZIF-8 = 1.10 g, RT = 1.88 g, PBS = 2.28 g).

The toxicity of all drugs was tested by observing behavioral changes after treatment and monitoring body weight (Figure 7D). After drug treatment, the groups did not show significant weight loss throughout the treatment course. In addition, the histological examination also showed no significant systemic toxicity in each treatment group (Figure 7E), which is consistent with previous results. This benefit may stem from the fact that the hybrid membrane is biocompatible and provides excellent targeting power. In addition, ZIF-8 is pH-sensitive and releases the drug only in the tumor microenvironment, thereby greatly reducing the toxic side effects of systemic chemotherapy.

### 3.7 [Dbait-ADM@ZIF-8]OPM inhibits DNA repair *in vivo*

**FIGURE 8:**
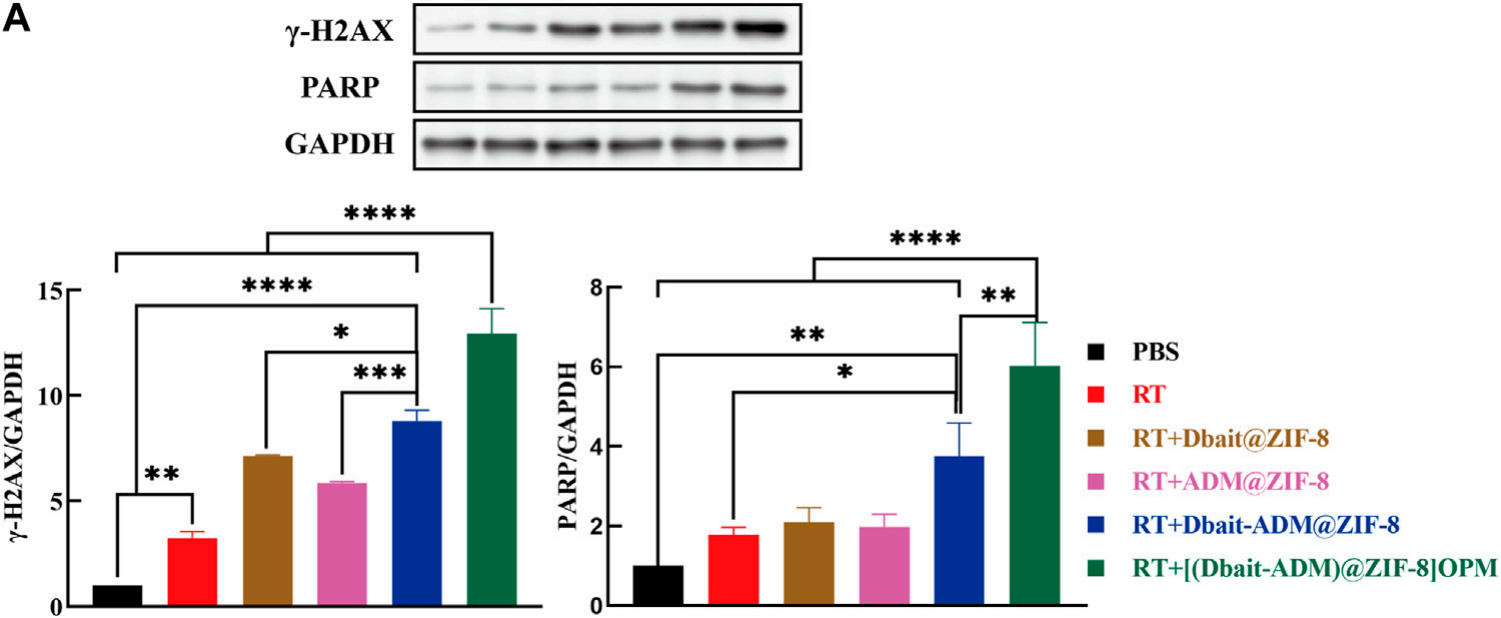
**(A)** Western blot analysis of γ-H2AX, PARP protein levels in OS organization.

We further elucidated the molecular mechanism of [Dbait-ADM@ZIF-8]OPM in the improvement of the effect of radiotherapy and chemotherapy in OS. Per the results of *in vitro* experiments, immunoblotting showed Dbait@ZIF-8, ADM@ZIF-8, and Dbait-ADM@ZIF-8 in tumor tissues in the treatment group; and the protein expression levels of γ-H2AX and PARP in tumor tissue in the [Dbait-ADM@ZIF-8]OPM treatment group were significantly higher than those in the control and RT groups. The expression level of the [Dbait-ADM@ZIF-8]OPM group was significantly higher than that of other groups (Figure 8). These results confirm that [Dbait-ADM@ZIF-8]OPM has excellent targeting ability and the drug is highly enriched in the tumor, thus giving the nanocarrier excellent anti-tumor effects.

## 4 Discussion

OS is a primary malignant bone tumor occurring in children and adolescents, mostly in the epiphysis of long bones, and is characterized by malignant spindle cells that produce osteoid and bone (Bacci et al., 2008; Zhu et al., 2018). The current comprehensive treatment model of “preoperative and postoperative adjuvant chemotherapy + surgical resection of tumor” has improved the 5-year survival rate of patients with OS from less than 20%–60% (Bernthal et al., 2012). However, in recent years, there has not been a breakthrough in the efficacy of treatment, and the main reasons for treatment failure are not only the difficulty of surgical resection, recurrence of OS, and lung metastasis but also the serious toxic side effects of chemotherapy, which is the main treatment modality, and the poor response of OS to radiotherapy (Schwarz et al., 2009; Harrison et al., 2018; Li and Zhang, 2018).

To improve the efficacy of RT on OS, this study used Dbait as a radiosensitizer to increase the sensitivity of SaOS-2 cells to RT (Liu et al., 2017). Previously, Dbait combined with radiotherapy and chemotherapy was shown to be effective in several radiotherapy- and chemotherapy-resistant tumors (Biau et al., 2016; Herath et al., 2016; Yao et al., 2016). Therefore, ADM, a first-line chemotherapeutic agent for Adriamycin OS, was chosen in combination with radiation therapy to explore its therapeutic effect on OS and its mechanism.

In this study, a novel targeted drug delivery system was developed using a combination of radiochemotherapy regimens for OS. The results of agarose gel electrophoresis showed that the best Dbait encapsulation efficiency was displayed at an N/P ratio of 12. [Dbait-ADM@ZIF-8]OPM (Figure 2) was successfully prepared, as demonstrated by the WB analysis, electron microscopy, particle size, Zate, and XRD experiments (Ren et al., 2014; Zeng et al., 2019). The system uses ZIF-8 loaded with ADM and Dbait. ZIF-8 has a large internal space and excellent pH sensitivity, which was also verified in this study (Figure 3). The drug release rate was greatly enhanced at pH 5, which provided a layer of assurance for the stable release of the drug loading system in the tumor microenvironment. This property endows the nanocarriers of the ZIF-8 series with certain targeting properties. This is because the 2-MI linkage chain in ZIF-8 can be protonated in the acidic environment of tumor cells (pH = 4.5–6.5), thereby disrupting the coordination between the zinc ion and the imidazole ring, leading to the gradual degradation of the ZIF-8 structure and the release of the drug (Sun et al., 2012).

However, reliance on ZIF-8’s targeting is insufficient to achieve immune escape and efficient enrichment in tumors. This was also confirmed in this study (Figures 4, 6); only a small fraction of ZIF-8 alone entered the tumor cells in the *ex vivo* experiments, which led to its efficacy not reaching the expected levels. Bionanomaterial cell membrane coating technology has great potential in the field of medicine, including tumor therapy (Yu et al., 2018). Nanotechnology can mold cell membranes to nanoscale dimensions and combine the natural biological functions of the relevant cell membranes with the properties of nanocarriers to achieve simultaneous multiple therapeutic effects (Chen et al., 2020). The OS-PLT hybrid membrane developed in this study has good biocompatibility, which confers excellent targeting ability to nanocarriers, enables immune escape, and allows efficient drug enrichment in tumor cells. This can be attributed to the good homologous targeting of OCM and the release of “don't eat me” signals from CD47 on the PM surface, which reduces the phagocytosis of the nanocarriers inside the membrane by macrophages (Rao et al., 2016; Zhu et al., 2016; Li et al., 2017). In addition, the PLM surface also expresses unique surface receptors represented by P-selectin, which dynamically adheres to damaged blood vessels and tumor cells (Lievens and von Hundelshausen, 2011; Kappelmayer and Nagy, 2017). These advantages not only prolong the *in vivo* circulation time of nanocarriers but also alleviate the toxic side effects, as demonstrated by the weight change and histological examination of nude mice in this study (Figures 7D,E).

Thus, [Dbait-ADM@ZIF-8]OPM exhibited excellent anti-tumor ability (Figures 5A–C; Figures 7A–C). The study hypothesizes that the high enrichment of Dbait and ADM in tumor cells is due to not only hybridized membranes and nanocarriers but also the synergistic effect of both drugs in the therapeutic mechanism. Under RT conditions, Dbait inhibits the repair of DSBs and exerts a radiosensitizing effect. The ability of Dbait to mimic DNADSBs and activate damage signaling kinase activity was confirmed by the analysis of γ-H2AX, PARP in tumor cells (Figures 5D, 8). The high drug loading of Dbait is due to DOTMA, a cationic lipid, which is often used as a transfection agent for nucleic acids (Bhavsar et al., 2012). The DOTMA cationic ammonium head group is effectively attracted to negatively charged DNA and maintains the stability of the nanocarrier (Mohammadi et al., 2019). In addition, ADM binds to DNA and blocks DNA synthesis *via* embedding (Fu et al., 2016). The above mechanism, in addition to directly killing tumor cells, greatly limited their proliferation, thereby achieving unexpected efficacy. Thus, this study represents a breakthrough in the treatment of OS. Considering that the current experiments are still superficial in terms of mechanism research and lack more in-depth findings, we hope that our findings can encourage further exploration of the underlying molecular mechanisms.

## 5 Conclusion

In this study, we developed a novel [Dbait-ADM@ZIF-8]OPM with excellent targeting ability to efficiently deliver Dbait and ADM to SaOS-2 cells for the synergistic treatment of OS with significant efficacy. Based on its excellent biocompatibility, it has no significant toxicity to normal tissues during the treatment process. The study’s results demonstrate that [Dbait-ADM@ZIF-8]OPM is a viable option to achieve integrated radiotherapy treatment for OS.

## Data Availability

The original contributions presented in the study are included in the article/Supplementary Material, further inquiries can be directed to the corresponding authors.
