# Supplementary material for: Integrated radiochemotherapy study of ZIF-8 coated with osteosarcoma-platelet hybrid membranes for the delivery of Dbait and Adriamycin
Source: Front Bioeng Biotechnol. 2023 Feb 17;11:1147064. doi: 10.3389/fbioe.2023.1147064 (PMC9981937; doi:10.3389/fbioe.2023.1147064)
Supplement: Supplementary file 1 [file DataSheet1.docx]

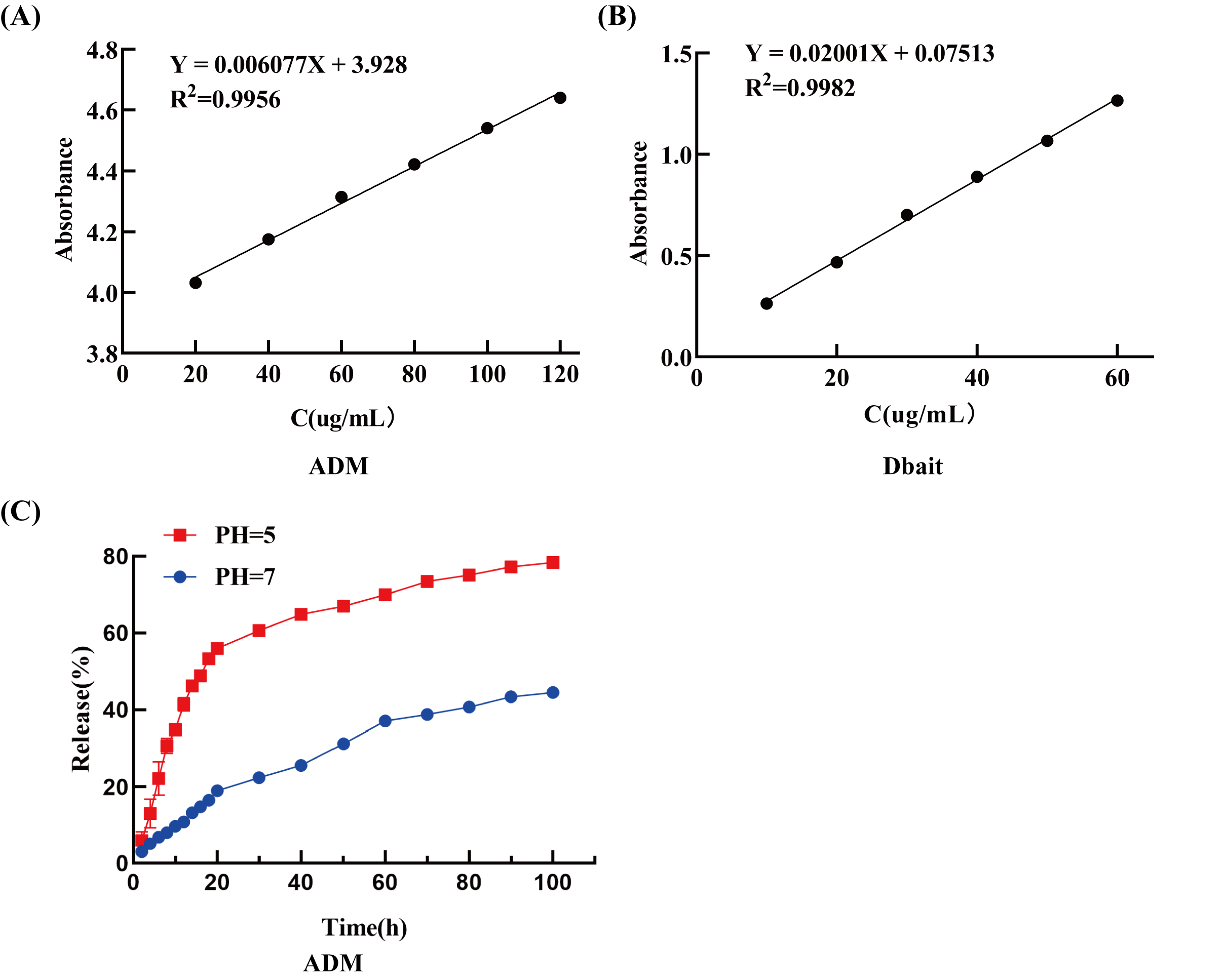


Fig.S1.Release standard curves for (A) AMD and (B) Dbait. (C) Cumulative release rate of ADM in PBS at pH=5, 7 in [ADM@ZIF-8]OPM.
